# Myocardial ischemia with left ventricular outflow obstruction

**DOI:** 10.1186/1749-8090-4-51

**Published:** 2009-09-17

**Authors:** Aron F Popov, Christian Bireta, Jan D Schmitto, Dieter Zenker, Martin Friedrich, Kasim O Coskun, Ralf Seipelt, Gerd G Hanekop, Friedrich A Schoendube

**Affiliations:** 1 Department of Thoracic and Cardiovascular Surgery, University of Göttingen, Germany; 2 Division of Cardiac Surgery, Department of Surgery, Brigham and Woman's Hospital, Harvard Medical School Boston, MA, USA; 3 Department of Anaesthesiology, Emergency and Intensive Care Medicine, University of Göttingen, Germany

## Abstract

We report an unusual case of a 32-year old man who was treated for a hypertrophic obstructive cardiomyopathy (HOCM) with a DDD pacing with short AV delay reduction in the past. Without prior notice the patient developed ventricular fibrillation and an invasive cardiac diagnostic was performed, which revealed a myocardial bridging around of the left anterior descending artery (LAD). We suspected ischemia that could be either related to LAD artery compression or perfusion abnormalities due to AV delay reduction with related to diastolic dysfunction.

## Case report

We are reporting on a 32-year-old patient who had been treated for a prolonged period of time for symptoms of HOCM. However, the diagnosis of a flow acceleration and pressure gradient in the outflow tract had only been made by echocardiography (ECHO) up to that point. In 2004, a 2-chamber pacemaker with a short AV conduction time (70 ms) was implanted for a left precordial repolarization abnormality and to lower the pressure gradient between the ventricle and outflow tract. Freedom from symptoms was not actually achieved with this treatment. In February of this year, the patient was found in a non-responsive state following what had most likely been a period of complete well-being. Emergency cardiovascular resuscitation was not started immediatly, however, and the interval before resuscitation was started was approximately 5 minutes. When the emergency medical services arrived, the patient had no pulse of his own, while the pacemaker continued to work. After a short period of cardiopulmonary reanimation, a status of ventricular fibrillation was reached. At this point, a single defibrillation of 200 joules was applied and spontaneous circulation was established once again. The patient was only taking inadequate gasping breaths; as a result, he was intubated immediately. A low dose of adrenaline was administered due to hypotonic circulation; the patient then became hemodynamically stable. Upon admission to our clinic, cerebral imaging was immediately ordered; this showed no intracerebral bleeding, ischemia or edema. Neuroprotective hypothermia therapy was also immediately introduced for a period of 48 hours. Echocardiography revealed severe left ventricular hypertrophy with normal left ventricular ejection fraction (EF > 60%) and normal dimensions. Additionally, the ECHO exhibited nearly complete obstruction of the the left ventricular outflow tract (LVOT) by the hypertrophied septum including a systolic anterior motion phenomena (SAM) by the anterior leaflet of the mitral valve.

**Figure 1:**
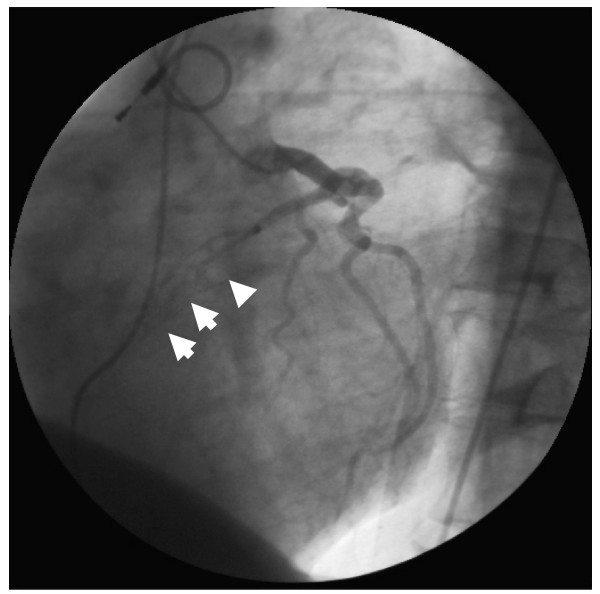
**Preoperative angiogram showing the appearance of the muscular bridge over the left anterior descending artery during systole**.

**Figure 2:**
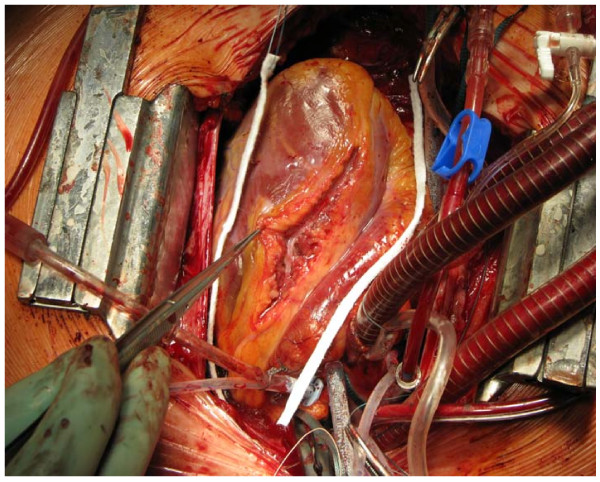
**Intraoperative view during the reconstruction of LAD**.

**Figure 3:**
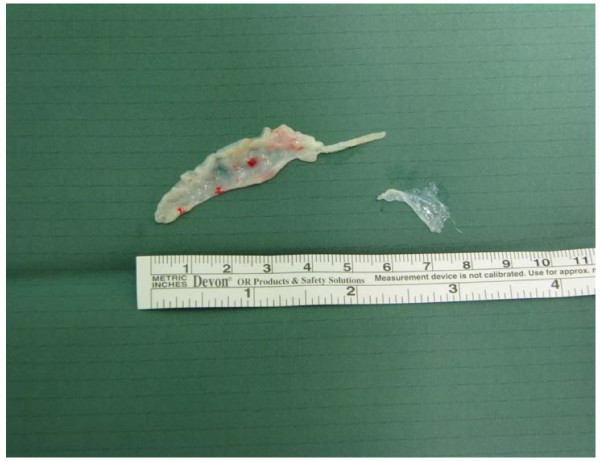
**Excised subaortic membrane**.

The subsequently performed cardiac catheterization revealed a non-typical obstruction involving the ventricle in the ventriculography. There was also a subtotal muscle bridge of the LAD (Fig. 1) leading to a virtually obstructive systolic vascular pattern. After stabilizing all parameters, the patient was finally transferred to our section for a surgical myectomy for HOCM. The LAD was unroofed from its myocardial bridge (Fig. 2). However, intraoperative findings surprisingly showed a membranous subaortic stenosis (Fig. 3) with consecutive left ventricular hypertrophy.

**Figure 4:**
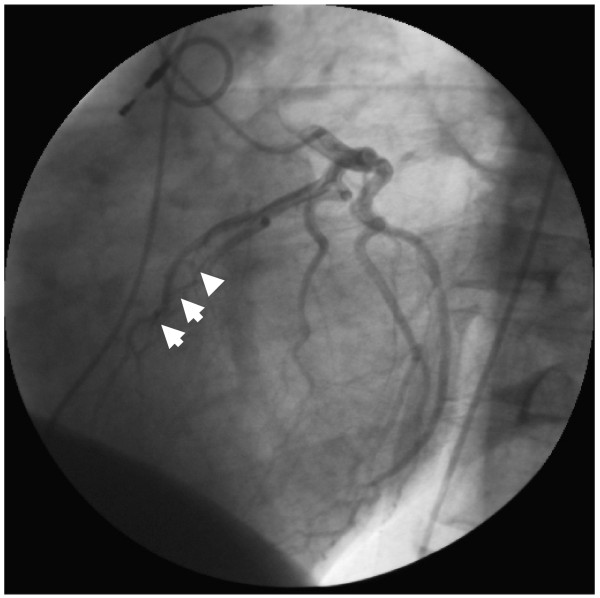
**Postoperative angiogram showing patent left anterior descending artery during systole, after incision muscular bridge and reconstruction**.

The patient survived the operation and his symptoms were relieved. The postoperative course was uneventful and postoperative coronary angiogram showed good resolution of the muscle bridge in the patient (Fig. 4).

## Discussion

HOCM is a complex and heterogeneous disease of cardiac muscle with a variety of functional, morphologic and clinical manifestations and is associated with various clinical presentations ranging from complete absence of symptoms to sudden, unexpected death. The overall prevalence of hypertrophic cardiomyopathy (HCM) has been estimated to average between 0.02% and 0.2% of the population [1]. The established medical therapy for patients with HOCM is a trial of large doses of beta-blockers, calcium channel antagonists, or disopyramide, either alone or in combination. Operation is a well established procedure with more than 40 years of experience and has been the gold standard therapy for those severely symptomatic patients with fixed or inducible gradients who are intolerant of these medications or unresponsive to them [2-6]. In addition, alcohol septal ablation, well-accepted as a potentially curative therapy for HOCM-patients refractory to maximal medical therapy, is still considered innovative and becomes widely accepted [7-10].

Additionally, permanent dual-chamber (DDD) pacing has been proposed as an adjunct treatment to reduce symptoms in markedly symptomatic patients with HOCM [11,12]. Several early observational and uncontrolled studies have suggested that dual chamber pacing has a benefit in patients with HOCM. The beneficial effects were alteration of the myocardial activation sequence inducing dyssynchronous ventricular activation and paradoxical septal movement, negative inotropic effects, and alteration of mitral valve leaflet excursion [13-15]. However, there are numerous studies of dual chamber pacing that have yielded conflicting results. These studies have investigated the beneficieal effects of pacing on gradient, pacing on gradient, symptoms, and quality of life, but have failed to make a definitive statement concerning the risk of sudden death at the present time. Finally, it is well known that in patients with HOCM supraventricular tachycardias are common and several factors may make these patients susceptible to ventricular fibrillation [15]. Furthermore, in our case the coronary angiogram reveals a left anterior descending coronary artery bridge. Myocardial bridging with compression of epicardial vessels occurs in 30-50% of adults with HOCM [16]. The role of myocardial bridging in ischemia and sudden death in patients with HOCM remains controversial [17]. Regardless, a poorer outcome of HOCM combined with bridging has been outlined [18].

Interestingly, the operation of our patient revealed a subaortic obstruction in the form of subaortic membrane, which has a congenital basis and is responsible for 8% to 20% of cases of congenital left ventricular outflow obstruction (LVOT) [19]. In most instances, the lesion progresses to produce LVOT obstruction during childhood and adolescence, while in some instances it does not [20]. However, there is little information about this lesion in adults.

Finally, it remains unclear what clinicopathological reasons induced the sudden cardiac death. It is possible, that the dual chamber pacing with AV delay reduction and/or the left artery descending bridge induced ischemia which leads to ventricular fibrillation with cardiopulmonary resuscitation, otherwise it is also possible that only the LVOT obstruction lead to ventricular defibrillation. The benefit of ICD treatment has been been well established among patients at high risk of sudden cardiac death in secondary and primary prevention seetings. However, in this case the myocardial bridging and left ventrucular outflow obstruction was surgically controlled. Therefore, we renouced to implantate an ICD.

Thomson et al. have demonstrated the mechanisms of myocardial ischemia in HOCM-patients with magnetic resonance imaging (MRI). Possibly, this promising diagnostic option becomes routinely available for patients with HOCM and signs of ischemia.

These patients are a technical challenge and should be treated by very experienced surgeons, cardiologists, and anaesthetists in specialized institutions.

## Conclusion

On the basis on this case, dual chamber pacing can not regarded as primary treatment modality for left ventricular obstruction. For a select subset of patients with refractory LVOT obstruction, DDD pacing with a short atrioventricular (AV) delay may be of benefit or represent a therapeutic option for some elderly patients with HOCM, particularly those who reject operation/alcohol septal ablation or do not have access to experienced surgeon and cardiologists in specialized institutions. However, short delay DDD-pacing is obviously not acceptable in patients with an obstructive subaortic membrane.

## Consent

Written informed consent was obtained from the patient for publication of this case report and any accompanying images. A copy of the written consent is available for review by the Editor-in-Chief of this journal.

## Competing interests

The authors declare that they have no competing interests.

## Authors' contributions

AP and FS are members of the surgical team, conceived of the study, and participated in its design and coordination. CB, JS, and DZ were involved in the postoperative treatment and were participated in its coordination. RS, MF, and KC added important comments to the paper and helped to draft the manuscript. GH was the anaesthetist involved in theatre and in intensive care unit and helped to draft the manuscript. FS co-wrote the manuscript. All authors have read and approved the final manuscript.
